# Understanding how unhealthy food companies influence advertising restrictions

**DOI:** 10.1371/journal.pmed.1003742

**Published:** 2021-09-02

**Authors:** Stanton A. Glantz

**Affiliations:** San Francisco, California, United States of America

Because of the growing global importance of noncommunicable diseases (NCDs), including cardiovascular disease, cancer, chronic respiratory disease, and diabetes, the United Nations adopted the 2011 Political Declaration of the High-Level Meeting of the General Assembly on the Prevention and Control of Non-Communicable Diseases [1]. The declaration recognized that NCDs are “linked to common risk factors, namely, tobacco use, alcohol abuse, an unhealthy diet, physical inactivity, and environmental carcinogens.” In contrast to infectious disease, exposure to these risk factors is increased by corporations actively and consciously working to increase the consumption of unhealthy products to maximize profits [2]. As a result, in contrast to controlling nonsentient infectious diseases, public health interventions to reduce NCDs have to anticipate and counter conscious opposition from these corporate interests.

Advertising is a key vector used to promote unhealthy products, and there is a long history of efforts to ban or restrict advertising as a key element of tobacco control [3]. Ulucanlar and colleagues [4] used the substantial literature on how the tobacco industry opposes advertising restrictions (and tax increases) to develop the policy dystopia model (PDM) as a theoretical framework to understand this opposition. They found that the tobacco industry used “discursive” (argument based) and “instrumental” (action based) strategies to work through third parties and front groups to argue that proposed public health policies will not only fail but also lead to widely dispersed adverse social and economic consequences. The real motivation for industry opposition—maintaining markets and profits—is rarely, if ever, mentioned. While based on experience in just 2 policy areas (tobacco advertising restrictions and taxation) in high-income countries, the PDM has been successfully applied to other tobacco control issues and in low- and middle-income countries [5–8]. In this issue of *PLOS Medicine*, Lauber and colleagues [9] apply the PDM to analyze food industry opposition to London’s 2018 proposed ban of advertising of foods high in fat, sugar, and/or salt (HFSS) across Transport for London (TfL), a major advertising channel.

They used the Freedom of Information Act to obtain industry responses to the London Food Strategy Consultation, correspondence between officials and key industry actors, and information on meetings. Except for some smaller businesses, most food and advertising industry respondents opposed the proposed advertising restrictions. Consistent with the PDM, many of these comments contested the evidence supporting the ban, promoted ineffective voluntary approaches, exaggerated costs of implementation and to society, and underplayed benefits of the advertising ban. Some industry comments also hinted at potential legal action, although none was filed during the period reported in the paper (through April 2021). Significantly, the adverse effects on food industry profits were not among the arguments that the industry raised against the proposed advertising ban.

Unfortunately, the Greater London Council did not publish a detailed draft policy as part of the consultation, so it was not possible to assess how effective industry lobbying was in terms of impacting the policy. Health interests should press for publication of draft policies in order to both be able to comment on (and support, when appropriate) specific policies as well as assess the extent of government deference to industry in proposing initial policies or industry influence in weakening good policies.

Creating a public health policy is only the first step; it is also important that the policy be implemented and enforced. Lauber and colleagues [9] report that of 81 exception applications, TfL accepted two-thirds (54). As Lauber and colleagues note, the TfL staff members responsible for enforcing the policy and granting exemptions are from TfL’s advertising team and also responsible for meeting advertising revenue targets. This potential conflict of interest issue is worthy of future research.

In addition, Lauber and colleagues only assess comments from industry that were critical of the rule, even though some businesses supported the rule (their S2 Table in [9]). As they report, these businesses tended to be smaller local businesses, whereas the critics tended to be major multinational corporations and their agents and allies. The authors do not assess the arguments submitted by the public health community at all. These issues are also worth further exploration, because doing so could guide public health authorities and advocates on how to argue for and mobilize business support for public health interventions.

In 2018, the UN held another high-level meeting to assess progress in implementing the 2011 declaration [1] and adopted another declaration that included more specifics, including a commitment “to further reduce the exposure of children to and impact on them of the marketing of foods and beverages high in fats, in particular saturated fats and *trans*-fats, sugars, or salt” [10, clause 44(e)]. Lauber and colleagues [9] provide important insights to guide the implementation of this recommendation as well as contributing to the developing literature on corporate determinants of health [2]. In particular, corporations that profit by selling unhealthy products are one of the vectors for NCDs just as mosquitoes are the vector for infectious diseases. Progress on reducing NCDs will require understanding, anticipating, and countering these corporations’ efforts to block effective NCD policies.

## Abbreviations

HFSS: high in fat, sugar, and/or salt
NCD: noncommunicable disease
PDM: policy dystopia model
TfL: Transport for London
